# Carcinoma of Prostate

**Published:** 1983-07

**Authors:** A. M. N. Gardner, N. Setakis

**Affiliations:** Department of Surgery, Torbay Hospital; Department of Surgery, Torbay Hospital


### Carcinoma of Prostate. Increased Mortality and Cardio-vascular Complications Associated with Oestrogen Therapy as Compared with Orchiectomy

**A. M. N. Gardner, M.Ch. and N. Setakis, MD.**
Department of Surgery, Torbay Hospital

The treatment of prostatic carcinoma, the second commonest carcinoma in the Western world, remains controversial although opinion has leaned towards oestrogens rather than orchiectomy unless the patient suffers a thrombo-embolic tendency.[9, 10]

We report here a retrospective study of two statistically comparable groups of patients: one treated with oestrogens (Stilboestrol) the other by orchiectomy.

### MATERIALS AND METHODS

A total of 226 cases admitted to two similar surgical services in a district hospital (Torbay) between 1967 and 1979 were studied.

On one service treatment was by subcapsular orchiectomy, on the other oestrogens were used. The two series were statistically comparable in respect of age (average in both was 75 years), acid phosphatase and urea levels. Biopsy was not routinely performed. Early cases without raised acid phosphatase were included only if calculi were excluded as a possible cause of prostatic nodularity. Cases with clinical evidence of distant metastases were excluded from the study. No patients were treated by radical prostatectomy, radiotherapy or cytotoxic therapy, nor was lymphography performed.

### RESULTS

At the mean age of our patients in both groups (75) in males the 5-year expectation is 70%:[11] 5-year survival in our orchiectomy patients was 60%, but in the oestrogen treated patients it was only 34%.

A total of 143 were still alive and available for follow up with an average age of 76 years (Table 1). These patients were interviewed to establish their present clinical condition. The causes of death in the remainder were ascertained through the General Practitioners and the Registrar General's records.

**Table 1**

| | NUMBER OF PATIENTS | TREATED BY ORCHIECT. | TREATED BY STILBOEST. |
|---|---|---|---|
| TOTAL | 226 | 106 | 120 |
| ALIVE | 143 | 80 | 63 |
| DIED | 83 | 26 (2 M.I.) | 57 (25 M.I.) |
| 5 YEAR | 89 | 52 | 37 |
| AVER. AGE | 76 | 76 | 75 |

Of the 120 patients treated with Stilboestrol (usually 9 mg daily), 54% were still alive; 108 started treatment 5 or more years previously and 37 were still alive. The 5 year survival rate was 34% (Table 2).

**Table 2**

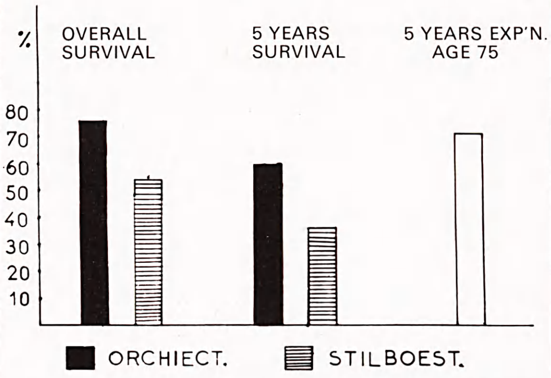

Of the 106 patients treated by orchiectomy three quarters were still alive; of these 87 were treated more than 5 years previously and of them 52 were still alive giving a 60% 5-year survival rate.

Of the total of 83 patients in the two groups who died, 26 had been treated by orchiectomy and 57 by Stilboestrol. Coronary thrombosis was the cause of death in 25 of those treated by Stilboestrol, and in only two of those treated by orchiectomy. In both groups most of the remaining deaths were from carcinomatosis (Table 3).

**Table 3**

REPORTED CAUSES OF DEATH

| | ORCHIEC. | STILBOES. |
|---|---|---|
| COR. THROMB | 2 | 25 |
| CARCINOMAT. | 22 | 31 |
| PNEUMONIA | 2 | - |
| GEN. SENILITY | - | 1 |

These figures indicate that a large number of patients treated by 9 mg Stilboestrol died from coronary thrombosis before the cancer killed them.

In our clinical follow up 143 patients were interviewed: 80 had been treated by orchiectomy and 63 by Stilboestrol.

Of the patients treated by Stilboestrol one in five (20%) started anti-hypertensive drugs shortly after starting treatment. The majority of them developed gynaecomastia, but only a small number of them complained of hot flushes (Table 4); this was in contrast to those treated by orchiectomy, the majority of whom suffered flushes, but none of them had been treated for hypertension and two only had mild gynaecomastia (Table 4).

**Table 4**

| | ORCHIECT. | STILBOEST. |
|---|---|---|
| NOCTURIA | ALL | ALL |
| GYNAECOM. | 2 | 59 |
| HOT FLUSH | 61 | 17 |
| HYPERTENSION | NONE | 25 |

### DISCUSSION

The original treatment of carcinoma of prostate was only to relieve the urinary obstruction and pain due to bone metastases,[3] and to this day many believe that in Stage I cases no treatment other than relief of obstruction is required.[9] Suppression of androgen activity by castration or oestrogens has been used in prostatic carcinoma since Huggins and Hodges[8] showed their efficacy, though curiously more recently the anti-androgen cyproterone acetate has proved disappointing.

Theoretically there might be some doubt whether orchiectomy alone is enough to keep the plasma androgens low since the adrenals remain as a source of androgens; however, Clark and Houghton[4] found the mean plasma testosterone level in 83 patients after orchiectomy to be 43 mg/100 ml, the same as in patients taking 5 mg of Stilboestrol daily. Other advantages of orchiectomy are that no medication is required and there is less gynaecomastia. No psychological problems were encountered in either group. Hot flushes were nearly universal and occasionally troublesome in the orchiectomy patients.

In common with Huggins[8] and Black et al.[1] we have found orchiectomy and oestrogen therapy to be equally effective in controlling symptoms, but in common with the Veterans Group,[13] Mellinger[11] and Heaney et al.,[7] we have demonstrated that oestrogens are less effective in prolonging life since they predispose to cardio-vascular accidents. Relevant here is our finding that one in five of our oestrogen-treated patients had required anti-hypertensive treatment.

In some clinics the dosage of Stilboestrol has been reduced to 1 mg and this has been considered to be about as effective in treating the carcinomas as 5 mg daily.[2] It has been alleged that this lower dose avoids the excess hazard of cardio-vascular deaths associated with the higher dose, this however seems to be most unlikely, since it is still 22 times the dose found to double the risk of thrombo-embolic disease in women to increase platelet stickiness and to lower anti-thrombin III levels.[5]

It is not yet possible to assess the ultimate value of the other methods of treating prostatic carcinoma, but certainly many patients whose disease reactivates after oestrogens or orchiectomy respond to Tamoxifen, radiotherapy or adrenalectomy; hypophysectomy is less effective.

### SUMMARY

A total of 266 patients with carcinoma of prostate were studied retrospectively; 120 had been treated by oestrogens, 54% survived to date of study and of 106 treated by orchiectomy 75% survived to date.

The 5-year survival rate with oestrogens was 34% and with orchiectomy 60%; 26% of the oestrogen group suffered coronary thrombosis but only 2% of the orchiectomy group; 20% of the oestrogen group, but none of the orchiectomy group, had been treated for hypertension.

### ACKNOWLEDGEMENTS

We would like to thank Mr. H. P. Guerrier, F.R.C.S., for permission to study a number of his patients.

### REFERENCES

1. BLACKEND, C. E., BYAR, D. P. and JORDAN, N. P. (1973) *Urology* **I**, 535.
2. BYAR, D. P. (1973) *Cancer* **32**, 1126.
3. CHISHOLM, G. D. and O'DONAGHUE, E. P. N. (1975) *Vitam.Horm.* **33**, 377.
5. COMMITTEE on SAFETY of DRUGS (1970) *British Medical Journal* **2**, 231.
6. FARGERHOL, M. K. and ABILDGAARD, V. (1970) *Scand. J.Haematol.* **7**, 10.
7. HEANEY, J. A., CHANG, H. C., DALY, J. J. and PROUT, G. R. (1977) *Journal of Urology* **118**, 283.
8. HUGGINS, C. and HODGES, C. V. (1941) *Cancer Res.* **1**, 293.
9. Lancet Editorial (1980) **2**, 1009.
10. LEADING ARTICLE (1977) *British Medical Journal* **2**, 752.
11. MELLINGER, G. T. (1977) *Recent Results Cancer Research* Part 60, 61.
12. Statistic Division, Prudential Life Insurance Company, London. Personal communication.
13. Veteran's Administration Co-operative Urological Research Group (1967) *Journal of Urology* **98**, 516.

